# Examining Mixtures of Disinfection By-Products: Rat Study Shows No Effects on Reproduction

**DOI:** 10.1289/ehp.123-A159

**Published:** 2015-06-01

**Authors:** Lindsey Konkel

**Affiliations:** Lindsey Konkel is a Worcester, MA–based journalist who reports on science, health, and the environment.

Disinfection of drinking water is regarded as one of the major public health achievements of the twentieth century, resulting in drastic reductions in diseases such as cholera and typhoid fever.^1^ However, potentially hazardous disinfection by-products (DBPs) can form when water treatment chemicals interact with other compounds in the water. In this issue of *EHP*, investigators assess the reproductive toxicity of a mixture of chlorination DBPs in rats.^2^

Some animal toxicity and human epidemiological studies have suggested that individual chlorination DBPs may be associated with an increased risk of birth defects, spontaneous abortion, delayed puberty, and reduced sperm quality.^3^^,^^4^^,^^5^ Other studies have shown no such associations.^5^ Little research has been conducted on the reproductive toxicity of these chemicals in mixture,^6^ which reflects the most realistic exposure. “The real world is a combination of all these chemicals,” says senior study author Jane Ellen Simmons, a toxicologist at the U.S. Environmental Protection Agency (EPA) National Health and Environmental Effects Research Laboratory.

**Figure:**
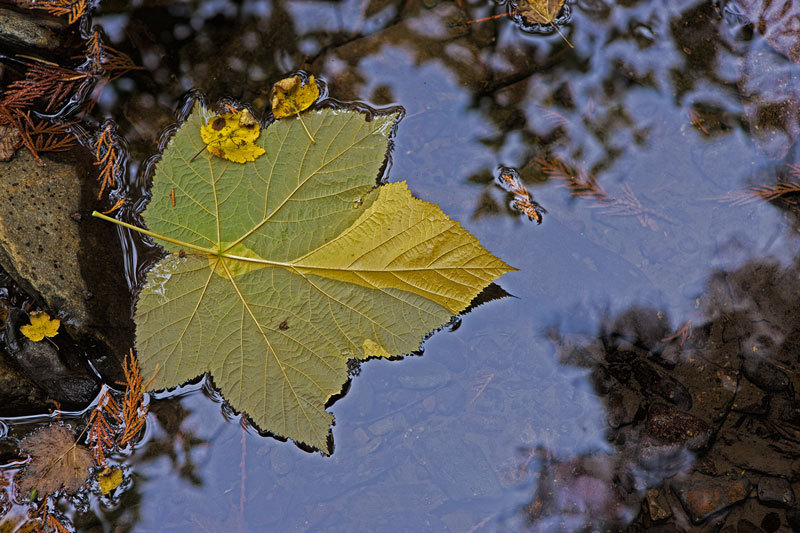
Natural organic matter in source waters combines with disinfectants such as chlorine to produce potentially toxic DBPs. © Gregory Johnston/Shutterstock

More than 600 unique DBPs have been identified,^5^ with trihalomethanes (THMs) and haloacetic acids (HAAs) the most prevalent DBPs found in chlorinated water. The EPA regulates four THMs and five HAAs (as well as two by-products formed by other disfection methods) as suspected human carcinogens.^7^ Rather than setting maximum contaminant levels for individual chemicals, the agency regulates total THMs and total HAAs.^2^

In the current study, Simmons and colleagues assessed the reproductive health effects of a mixture of the nine regulated chlorination DBPs across three generations of rats. Throughout pregnancy and weaning, dams drank water containing 500, 1,000, or 2,000 times the maximum contaminant levels of total THMs and total HAAs allowed under current drinking water regulations. The offspring (or F_1_ animals) continued exposure after weaning and through the births of their own litters (F_2_ animals), which were examined at birth and 6 days of age.

The researchers reported no adverse effects on fertility, pregnancy, pup survival, or birth weight at any dose in F_1_ animals, and no effects on survival or birth weight in F_2_ animals.^2^ This could suggest that the reproductive and developmental outcomes observed in previous studies may not be due to the regulated DBPs, says Susan Richardson, an environmental chemist and professor at the University of South Carolina, who was not involved in the study. Instead, she says, “There’s a possibility that some unregulated water disinfection by-products could be responsible for those earlier associations.”

Both female and male F_1_ offspring showed a significant delay in the onset of puberty at the two highest exposure levels. At the 2,000× level, puberty was delayed by 5.8 days in females and 5.7 days in males. Rats generally reach puberty at about 5–7 weeks of age.^2^

In F_1_ male offspring, the researchers found that testosterone levels in the testis were reduced by 50% in the 2,000× exposure group, compared with controls. They also observed decreased sperm motility and nipple retention in F_1_ males. Nipple retention in males may signal impaired androgen production; although rats of both sexes begin developing mammary tissue *in utero*, androgen production causes nipple development to regress in males by birth.^2^

Although birth weight was unaffected, F_1_ offspring exposed to the highest doses tended to weigh less later in life, compared with controls. This may have been due to the rats drinking less water at the 1,000× and 2,000× exposure levels—possibly because of the taste of the chemicals.^2^ “It’s not clear what the implications of these findings are,” says lead study author Michael Narotsky, a research toxicologist at the National Health and Environmental Effects Research Laboratory.

“The findings are consistent with a modest antiandrogenic effect,” says Paul Foster, chief of the Toxicology Branch at the National Institute of Environmental Health Sciences, who was not involved in the study. However, the authors suggest that reduced water intake at the highest doses may have played a role in delayed puberty as well as in body weight, and the lower body weights at these doses may, in turn, have contributed to nipple retention and compromised sperm motility.^2^

Simmons hopes the team’s research can provide a useful framework for determining health effects of complex mixtures. “Looking at a defined subset of chemicals within an environmentally realistic mixture provides a very powerful tool for determining which chemicals in a mixture may be driving risk,” she says.

“Water disinfection is vitally important,” Richardson adds. “Studies like this help us to determine the safest possible processes to use.”
